# Overhauser Dynamic Nuclear Polarization Enables Single Scan Benchtop ^13^C NMR Spectroscopy in Continuous-Flow

**DOI:** 10.1021/acs.analchem.4c03985

**Published:** 2025-02-21

**Authors:** Johnnie Phuong, Billy Salgado, Tom Labusch, Hans Hasse, Kerstin Münnemann

**Affiliations:** a Laboratory of Engineering Thermodynamics (LTD), RPTU Kaiserslautern, Erwin-Schrödinger-Straße 44, 67663 Kaiserslautern, Germany; b Laboratory of Advanced Spin Engineering—Magnetic Resonance (LASE-MR), RPTU Kaiserslautern, Gottlieb-Daimler-Straße 76, 67663 Kaiserslautern, Germany

## Abstract

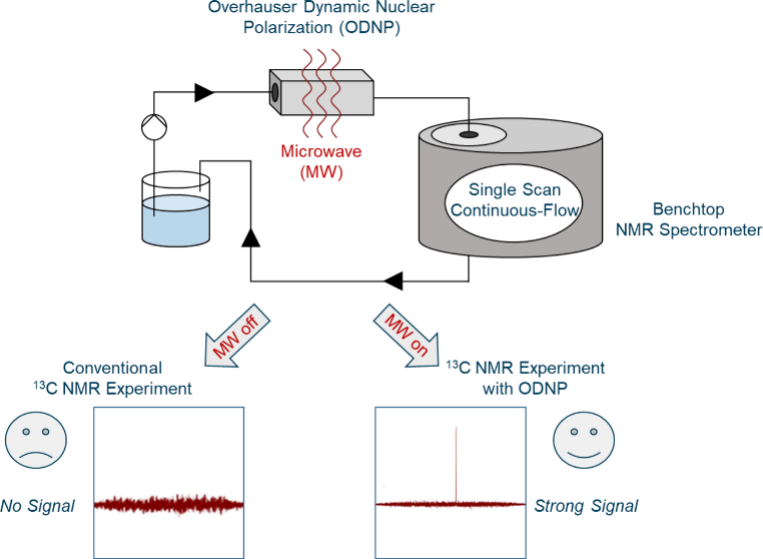

Benchtop ^13^C NMR spectroscopy is highly attractive for reaction and process monitoring. However, insufficient premagnetization and low signal intensities largely prevent its application to flowing liquids. We show that hyperpolarization by Overhauser dynamic nuclear polarization (ODNP) can be used to overcome these problems, as ODNP operates on short time scales and results in strong ^13^C signal enhancements. Benchtop ^13^C NMR spectra with ODNP enhancement acquired in continuous-flow are reported here for the first time. We have investigated two ODNP approaches: direct ODNP, which transfers the polarization of unpaired electrons to ^13^C nuclei via direct hyperfine coupling, and indirect ODNP, in which the electron polarization is first transferred to ^1^H nuclei before a polarization transfer pulse sequence finally transfers the polarization to the ^13^C nuclei. Experiments were carried out for three pure solvents and a mixture for different flow rates. The results show significant ^13^C signal enhancements for both approaches. However, their performance varies for different substances, depending on the strength and type of the hyperfine interaction as well as on the relaxation properties, but by selecting a suitable approach, good single-scan ^13^C NMR spectra can be obtained with benchtop NMR, even at high flow rates.

## Introduction

Nuclear magnetic resonance (NMR) is a highly attractive analytical technique for reaction and process monitoring due to its ability to noninvasively analyze complex chemical or biological mixtures.^1−6^ Benchtop NMR spectrometers are of great interest as they combine many advantages: Due to the use of permanent magnets, they are robust and compact, and they have low acquisition and operating costs.^7−14^ However, the low spectral resolution of benchtop spectrometers often leads to peak overlap, especially in ^1^H NMR, which makes it difficult to interpret the spectra. Mathematical methods (e.g., peak deconvolution^15^ or quantum mechanical modeling^16^) are available to analyze overlapping peaks, but expert knowledge is often required to obtain good results.

In many cases, the problem of overlapping peaks can be circumvented by using ^13^C NMR, which has a high chemical shift dispersion. However, also this method has drawbacks, in particular a low signal-to-noise ratio (SNR) and a slow magnetization build-up, due to long spin–lattice relaxation times *T*_1_ of ^13^C nuclei. This leads to extended acquisition times of ^13^C spectra for quantitative evaluation and makes it impossible to use the method for studies of kinetic effects, unless they are very slow. In addition, a further problem arises when the method is used to investigate flowing samples: If the flow rate is too high, the polarization build-up in the premagnetization volume becomes insufficient.^7,17^ This is particularly troublesome in benchtop NMR spectroscopy, where the premagnetization volume is typically small. These problems have so far prevented the use of ^13^C NMR for online monitoring of kinetic effects with flow benchtop NMR. We have shown that these problems can be partially overcome by using polarization transfer techniques, which transfer the polarization from ^1^H nuclei to ^13^C nuclei, since the ^13^C signal is enhanced and the polarization build-up depends on the shorter *T*_1_ time of the protons.^18^ However, the scope of this technique is severely limited.

The premagnetization issue could in principle be solved by the use of paramagnetic relaxation enhancement (PRE) agents.^19,20^ Immobilized PRE agents can be positioned in the flow path in front of the NMR coil, enabling a much faster premagnetization of the molecules and thus facilitating quantitative analysis at high flow velocities.^21−23^ Kircher et al.^24^ have successfully applied this technique to a 1 T benchtop NMR spectrometer. However, despite the accelerated polarization build-up, this method is not suitable for quantitative studies of kinetic effects with flow ^13^C NMR, because a large number of scans is still required to achieve a sufficient SNR. However, this problem can be solved by the application of NMR hyperpolarization methods which result in large signal enhancements.

Among various hyperpolarization techniques,^25−27^ such as parahydrogen induced polarization (PHIP)^28−33^ and optical pumping,^34−36^ Overhauser Dynamic Nuclear Polarization (ODNP) is particularly suitable for reaction and process monitoring.^37−41^ In ODNP, the high polarization of electron spins is transferred to surrounding nuclear spins via hyperfine coupling, enabling a maximum theoretical signal enhancement of 658 for ^1^H and 2640 for ^13^C nuclei, respectively.^42−51^ For ^13^C NMR spectroscopy, several studies have already demonstrated the potential of ODNP and have discussed the different hyperfine interactions in liquids.^52−60^ These studies were done on samples at rest and utilized dissolved radicals, which is not suitable for reaction and process monitoring, because radicals alter the sample and interfere with the NMR detection. In some works,^22,61−70^ it was demonstrated that the required radicals can be immobilized on matrices while largely retaining their ODNP performance. However, it should be noted that the ODNP performance of immobilized radicals is slightly lower than that of dissolved radicals due to their restricted dynamics. In ex-situ ODNP applications, the radical matrix is packed as a fixed bed in a flow cell, allowing flow-induced separation of the hyperpolarized fluid from the radical matrix and thus undisturbed NMR detection.^62,69,70^ This approach is especially well suited for continuously flowing samples, eventually allowing online monitoring of fast processes and reactions. Appropriate mobile ODNP setups compatible with benchtop NMR spectrometers are available which have been applied for benchtop ^1^H NMR spectroscopy under continuous-flow in previous benchtop studies.^69,71−74^ However, this approach has, to the best of our knowledge, not been used in ^13^C NMR studies.

The aim of this work is therefore to explore the possibilities of ODNP for benchtop ^13^C NMR spectroscopy in continuous-flow. Two approaches were studied: direct and indirect ODNP. In direct ODNP, the electron polarization is transferred directly to the ^13^C nuclei, which is the common approach in ODNP. In contrast, indirect ODNP transfers the electron polarization to the ^1^H nuclei in an intermediate step before the polarization is finally transferred to ^13^C nuclei via polarization transfer pulse sequences (e.g., INEPT or DEPT). Cheng et al.^75^ and Dey et al.^76^ have compared these approaches and refer to indirect ODNP as *J*-mediated and *t*-ODNP (transferred Overhauser DNP), respectively. Dissolved radicals were used in Cheng’s and Dey’s work and their measurements were performed on samples at rest, and not on flowing samples as it was done in the present work. The authors report that indirect ODNP resulted in larger enhancements than direct ODNP for some molecules, due to cancellation of site-specific positive (scalar coupling) or negative (dipolar coupling) enhancements in the same molecule.

In this work, both approaches were investigated for acetonitrile (ACN), chloroform (CF), and methanol (MeOH). No ^13^C enriched materials were used. The direct as well as indirect approach were also applied to a binary mixture of ACN and CF. Direct and indirect ^13^C ODNP experiments were performed on continuously flowing samples at different flow rates and the obtained signals (with respect to the signals obtained at thermal equilibrium) were compared. For the ^1^H to ^13^C polarization transfer, the pulse sequences PENDANT^77,78^ and refocused INEPT^+^ ^79^^,^^80^ were used. We have selected these two pulse sequences for ^1^H–^13^C polarization transfer for the following reasons: PENDANT detects both the original ^13^C polarization and the polarization transferred from ^1^H, which allows the detection of quaternary carbons; whereas INEPT only allows the detection of the ^13^C polarization resulting from the ^1^H transfer. The effect of both ^1^H–^13^C polarization transfer sequences on the net signal enhancement was investigated.

## Experimental Section

### Hardware and Experimental Procedure

**Figure 1:**
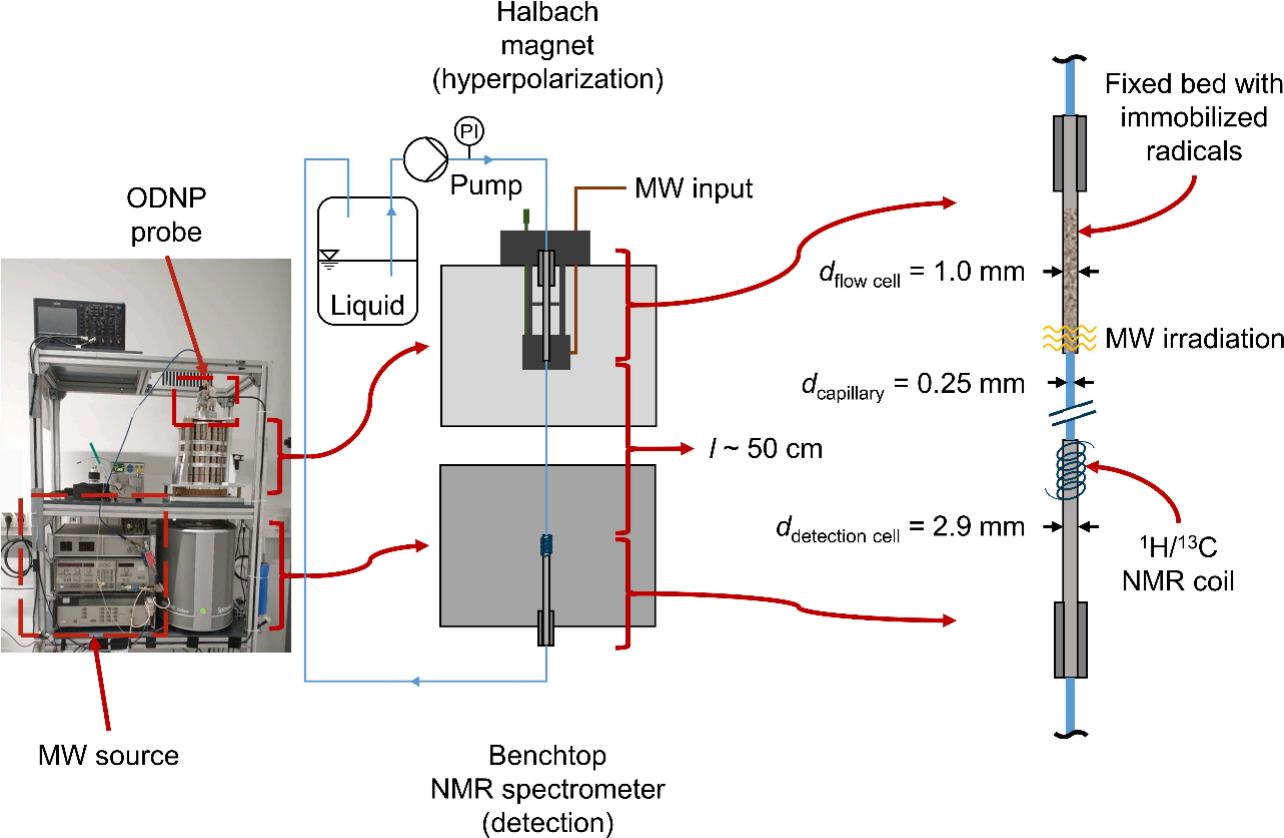
Photo and scheme of the experimental setup for continuous-flow ODNP experiment. MW: microwave.

The setup used for the continuous-flow ODNP experiments is illustrated in Figure 1. It was adapted from Kircher et al.;^69,70^ a detailed description of the setup is given in these references. The setup consists of two main parts: a Halbach magnet, in which hyperpolarization by ODNP is performed, and a benchtop NMR spectrometer for signal detection. Liquid was taken from a storage vessel (volume *V* = 100 mL) stored at room temperature and pumped through the setup by a double piston high pressure pump (WADose Plus HP, Flusys, accuracy: <3%). The pump speed was calibrated for a flow range of *V̇* = 0.5 to 10 mL min^–1^. The liquid first passes the Halbach magnet with the microwave (MW) resonator and the fixed bed containing the radical matrix. The ODNP probe was not thermostated; the temperature in the MW resonator is higher than the ambient temperature, for details see Kircher et al.^69^ As in the studies of Kircher et al.,^69,70^ the fixed bed was mounted in a PEEK tube with an inner diameter of *d*_flow cell_ = 1.0 mm. The length over which the MW field acted on the fixed bed in the MW resonator was 4 mm in height. The fixed bed was adjusted so that this MW active zone was at its end. After leaving the fixed bed, the liquid is fed to the benchtop NMR spectrometer through a PEEK capillary with an inner diameter of *d*_capillary_ = 0.25 mm.

The benchtop NMR spectrometer was from Magritek (Spinsolve Carbon) and had a magnetic field strength of *B*_0_ = 1.0 T, corresponding to a ^1^H Larmor frequency of ν_0_ = 42.5 MHz. The magnet of the benchtop NMR spectrometer was thermostated to ϑ = 28.5 °C. The maximum ODNP enhancement achievable with this setup is 220 for ^1^H and 880 for ^13^C, as the magnetic field strength used for detection is three times higher than the field strength used for hyperpolarization. In contrast to Kircher et al.,^69,70^ a PEEK tube with an inner diameter of *d*_detection cell_ = 2.9 mm was used as detection cell. The detection cell was positioned inside the benchtop NMR spectrometer so that the sensitive region of the NMR coil was located close to the expansion from 0.25 to 2.9 mm in order to minimize hyperpolarization losses caused by *T*_1_ relaxation. The total length of the line between the MW resonator and the detection cell was about *l* ≈ 52 cm. After leaving the NMR spectrometer, the liquid was recirculated to the storage vessel.

The flow rate was varied in the range between *V̇* = 0.5 and *V̇* = 7.0 mL min^–1^, corresponding to flow velocities of *v* = 0.17 to 2.38 m s^–1^ in the capillary between the Halbach magnet and benchtop NMR spectrometer. The pressure in the storage vessel was ambient, the pressure increase by the pump was between Δ*p* = 1 and 40 bar, and was indicated by the pressure gauge integrated in the pump (accuracy: 0.5%). ODNP experiments were performed at a MW frequency of *v* = 9.687 GHz. A MW power of *P* = 5 W was used to prevent MW-induced heating as much as possible. The MW irradiation was activated for at least 2 s before a NMR spectrum was acquired in the detection cell.

NMR experiments were controlled by the Spinsolve Expert software (Magritek). ^13^C NMR experiments with direct ODNP enhancement (referred to as ^13^C ODNP) were performed with an acquisition time of 1.6 s, 16 k data points, 1 scan and a 90° excitation pulse. ^13^C NMR experiments with indirect ODNP enhancement via the intermediate step of applying a polarization transfer sequence (PENDANT and refocused INEPT^+^) were also performed with an acquisition time of 1.6 s, 16 k data points and 1 scan (referred to as ^13^C ODNP PENDANT and ^13^C ODNP INEPT, respectively). The pulse sequences for both polarization transfer techniques are provided in the Supporting Information.

Furthermore, ^13^C NMR experiments performed at Boltzmann (thermal) equilibrium were used as a reference for calculating the ^13^C signal enhancements. The ^13^C NMR experiments without ODNP enhancement and in the absence of flow (referred to as ^13^C thermal) were performed with the same acquisition parameters except for the number of scans, which was 256 in order to obtain a sufficient SNR. The repetition delay was set to *t* = 120 s to ensure a full magnetization build-up of at least 5 times *T*_1,^13^C_. All ^13^C NMR experiments were performed with an inverse-gated decoupling sequence (WALTZ-16). Additional ^1^H NMR experiments with ODNP enhancement (referred to as ^1^H ODNP) were performed with an acquisition time of 0.4 s, 2048 data points, 1 scan and a 90° excitation pulse. The signal enhancements of the ^1^H ODNP experiments were calculated by using the signal obtained without ODNP at the same flow velocity which was also acquired with only 1 scan (referred to as ^1^H thermal). Furthermore, the spin–lattice relaxation times *T*_1,^1^H_ and *T*_1,^13^C_ of the molecules without and with contact to the immobilized radical matrix were determined with the inversion recovery experiment. NMR sample tubes with an inner diameter of *d* = 5 mm (Magritek) were used for the experiments without matrices, while NMR sample tubes with an inner diameter of *d* = 2.5 mm (Deutero) were used for the measurements with matrices. The latter were chosen in order to completely fill the sensitive region of the NMR coil with molecules that are in contact with the matrix. Only in this case ^13^C enriched solvents were used.

All experiments were repeated three times to calculate average signal integrals of the NMR peaks of component X in the respective NMR spectra, enhancements as well as standard deviations which are used for an error propagation. Details regarding the calculation of the error of the signal enhancement and the respective error bars are given in the Supporting Information.

The signal enhancement *E* of the ODNP experiments was calculated by scaling the spectra to the same noise level and by dividing the signal and the noise by the square root of the number of scans acquired, which was applied for the thermally polarized NMR spectra as well as for the NMR spectra with ODNP enhancement,^58,59^ see Equation 1.
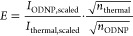
1

Here, *I*_ODNP, scaled_ denotes the integral of the scaled signal obtained with ODNP enhancement, *I*_thermal, scaled_ the integral of the scaled signal obtained at Boltzmann equilibrium, and *n* the number of scans. A correction for the receiver gain is not necessary since this parameter was kept constant in all ^1^H and ^13^C NMR experiments.

### Chemicals and Materials

Table 1 provides an overview of the chemicals used in this work. All chemicals were used without further purification. For preparing the binary mixture, a laboratory balance (XS6032S DeltaRange, Mettler Toledo, accuracy: ±0.01 mol mol^–1^) was used. ^13^C enriched chemicals were only used for the determination of the *T*_1,^13^C_ in contact with the radical matrix (marked with (^13^C) in Table 1) due to the low sample volume in the matrices.

**Table 1:** Chemicals Used in This Work^a^

Chemical	Formula	Supplier	Purity
ACN	CH_3_CN	Carl Roth	≥99.9%
ACN (^13^C)	^13^CH_3_CN	Cambridge Isotope Laboratories	≥99.9%
CF	CHCl_3_	Merck	≥99.0%
CF (^13^C)	^13^CHCl_3_	Cambridge Isotope Laboratories	≥99.9%
MeOH	CH_3_OH	Sigma-Aldrich	≥99.9%
MeOH (^13^C)	^13^CH_3_OH	Sigmal-Aldrich	≥99.9%

a Purity as specified by the supplier. ^13^C enriched chemicals are marked with ^13^C.

The immobilized radical matrix was synthesized in our laboratory and consists of the nitroxide radical glycidyl-oxy-tetramethylpiperidinyloxyl (TEMPO) immobilized on an amino-propyl-functionalized controlled porous glass (CPG) with a pore size of 50 nm. TEMPO was attached to the CPG via a polyethylene-imine (PEI)-linker (molecular mass of 25,000 g mol^–1^) and an intermediate linker 1,4-butanediol diglycidyl ether (BDGE). A detailed description of the properties of this immobilized radical matrix as well as its synthesis is given by Kircher et al.^70^ The concentration of the radicals on the matrix was approximately 37 mM.^70^

## Results and Discussion

In the following, the absolute signal integrals of the ^1^H ODNP, ^13^C ODNP as well as ^13^C ODNP PENDANT and ^13^C ODNP INEPT are shown for each studied substance as a function of the flow velocity. Negative signal enhancement due to dipolar coupling are also explicitly displayed as absolute values. For comparison, the corresponding results of the ^1^H and ^13^C thermal experiments are also reported. The numerical values as well as the signal enhancements achieved at each flow velocity are given in the Supporting Information. Furthermore, the results of the MeOH experiments are reported and discussed only in the Supporting Information.

The most interesting finding for MeOH is that ^13^C hyperpolarization can be only achieved by the indirect hyperpolarization scheme, which demonstrates its importance for ODNP experiments. Table 2 displays the maximum signal enhancements of ACN and CF as pure components as well as in the binary mixture obtained in the ^1^H ODNP, ^13^C ODNP, ^13^C ODNP PENDANT, and ^13^C ODNP INEPT experiment. The flow velocity at which the maximum signal enhancement was achieved is also reported. Good signal enhancement was obtained in all cases. Before entering into a detailed discussion of the results for each substance, we would like to address some general facts that are apparent in the experimental setup.

**Table 2:** Maximum Signal Enhancements *E* of the Molecules ACN and CF as well as in the Binary Mixture ACN + CF (*x*_ACN_ = 0.75 mol mol^–1^) for the Different ODNP Experiments^a^

	Maximum Signal Enhancement *E*			
	^1^H ODNP	^13^C ODNP	^13^C ODNP PENDANT	^13^C ODNP INEPT
ACN	5.2 (2.38)	8.2 (0.17)	5.1 (2.04)	5.6 (1.36)
CF	7.0 (1.70)	71.5 (0.85)	11.6 (1.02)	32.1 (1.02)
ACN + CF: ACN	4.0 (2.04)	11.5 (0.17)	4.6 (0.85)	-
ACN + CF: CF	4.8 (2.38)	31.5 (1.36)	15.0 (1.70)	-

a The corresponding flow velocities (in m s^–1^) at which the maximum signal enhancement was achieved are given in brackets. ^13^C ODNP INEPT experiments were not carried out for the binary mixture ACN + CF due to hardware failure.

**Figure 2:**
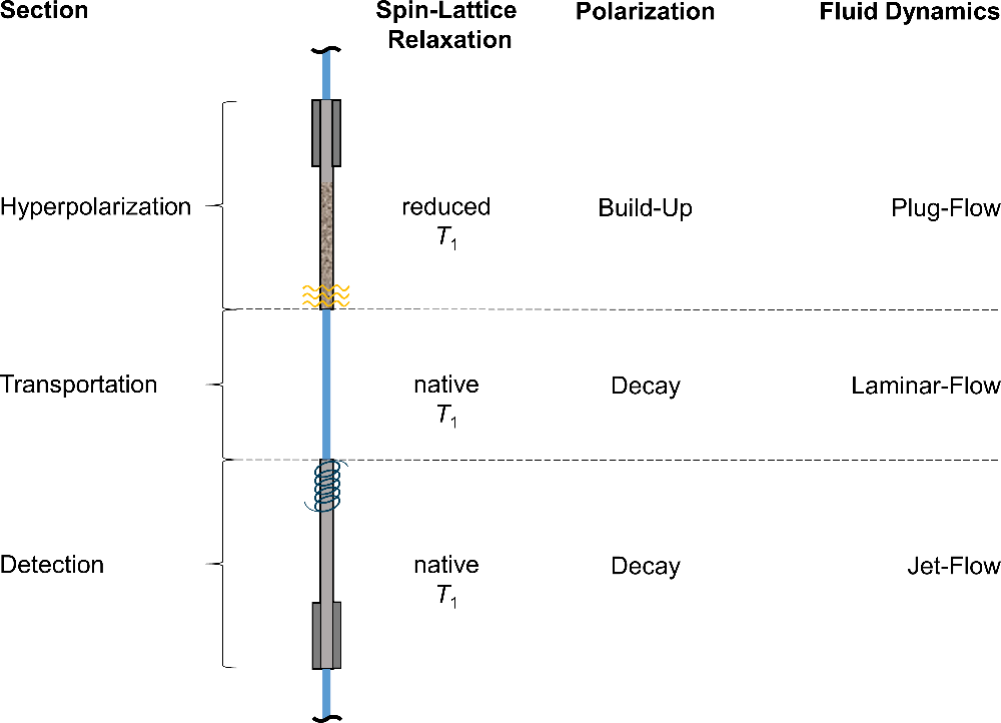
Illustration of the apparent spin–lattice relaxation, polarization, and fluid dynamics conditions in each section of the experimental setup.

Figure 2 illustrates the polarization conditions as well as the fluid dynamics in each section of the current experimental setup. In the Halbach magnet, the *T*_1_ time of the molecules is shortened by paramagnetic relaxation due to the contact with the radical matrix. The residence time of the liquid in the fixed bed (FB) located in the MW resonator is between τ_FB_ = 0.1 s and τ_FB_ = 0.01 s for the lowest and highest flow rates, respectively. These numbers were calculated assuming plug-flow and a void fraction of the fixed bed of ϵ = 0.26, which corresponds to that of the closest packing of spheres of the diameter we have used in the fixed bed. After the fluid has left the fixed bed, the ODNP hyperpolarization starts to decay at the rate of the native *T*_1_. The flow in the line connecting the fixed bed to the NMR detection cell (TL) is laminar. The mean transport time in that line is τ_TL_ = 3.1 s and τ_TL_ = 0.2 s for the smallest and highest flow rates, respectively. In the detection cell, the sudden expansion from *d* = 0.25 mm to *d* = 2.9 mm in combination with the high flow velocity leads to a jet-flow (jet with a small diameter in a larger cylindrical tube). Thus, near the axis of the detection cell, there is a fast flow of hyperpolarized fluid, whereas near the walls, only weakly polarized fluid flows in reverse direction and there are also zones in which the fluid is almost stagnant. This flow pattern severely reduces the observed enhancements. Lingwood et al.^68^ performed NMR imaging experiments to study such free jets with ODNP in a similar detection cell. It was shown, that the hyperpolarized fluid in the detection zone is surrounded by thermally polarized spins, which reduces the detected net signal enhancement. We have carried out a step experiment to characterize the flow regime in the detection cell, which are discussed in the Supporting Information. The mean residence time of the liquid in the active region of the NMR coil (DC; estimated length *l*_coil_ = 8 mm) is between τ_DC_ = 6.3 s and τ_DC_ = 0.5 s.

The analysis of the ODNP parameters (coupling, leakage, and saturation factors) was not the focus of this work. We focus here on the influence of the different *T*_1_ times in combination with the different residence times in the microwave cavity on the enhancement. In Table 3, the *T*_1,^1^H_ and *T*_1,^13^C_ values of ACN and CF without and with contact to the radical matrix are reported. As expected, a significant reduction of the *T*_1_ values due to paramagnetic relaxation is found. Furthermore, as expected, the *T*_1,^13^C_ values are significantly larger than the corresponding *T*_1,^1^H_ values for all substances. A short *T*_1_ during the matrix interaction is advantageous as it enables a faster and, hence, a complete hyperpolarization build-up in the Halbach magnet during continuous-flow. On the other hand, a long native *T*_1_ is desirable because it reduces hyperpolarization losses during the transport from the Halbach magnet to the benchtop NMR spectrometer.

**Table 3:** *T*_1,^1^H_ and *T*_1,^13^C_ Times Values of ACN and CF without (*T*_1,*i*_^0^) and with Contact to the Radical Matrix (*T*_1,*i*_^RM^) at *B*_0_ = 1 T^a^

	^1^H		^13^C	
Molecule	/s	/s	/s	/s
ACN	3.82 ± 0.05	0.10 ± 0.01	15.41 ± 0.30	0.54 ± 0.01
CF	5.25 ± 0.06	0.06 ± 0.01	21.69 ± 0.70	0.87 ± 0.06

a Mean value and standard uncertainty from three identical experiments are reported. For ACN, the  and  values refer to the CH_3_-group.

The ^13^C thermal signal cannot be measured in continuous-flow, so this signal was measured for a stagnant fluid with 256 scans. As a result, the ^13^C thermal experiments are not affected by insufficient polarization build-up and back-mixing effects due to the jet-flow. In contrast, all direct and indirect ODNP experiments are strongly influenced by the jet, since hyperpolarized and thermally polarized molecules are detected simultaneously. This fact results in lower detected net signal enhancements especially for ^1^H, where the thermal and hyperpolarized signals are of opposite sign and cancel each other out. For ACN, lower signal enhancement values were obtained compared to Kircher et al.^69^ using a 0.25 mm capillary for detection in which no jet-flow occurred. Moreover, due to the high radical concentration on the radical matrix and the fact that we chose a MW power of only *P* = 5 W (to reduce heating effects), the electron spin transitions were not fully saturated. This further reduces the measured signal enhancement. Furthermore, since the hyperpolarized sample is transferred from 0.35 to 1.0 T, a penalty factor of roughly 2.9 for the signal enhancement must also be considered. Another effect can reduce the enhancement further: For signal enhancements that originally have a negative sign (dipolar coupling), the signal can be canceled by a positive thermal signal. These effects greatly reduce the observed enhancements compared to the theoretically possible enhancements. Thus, the obtained enhancements are largely underestimated.

### ODNP Experiments with ACN

**Figure 3:**
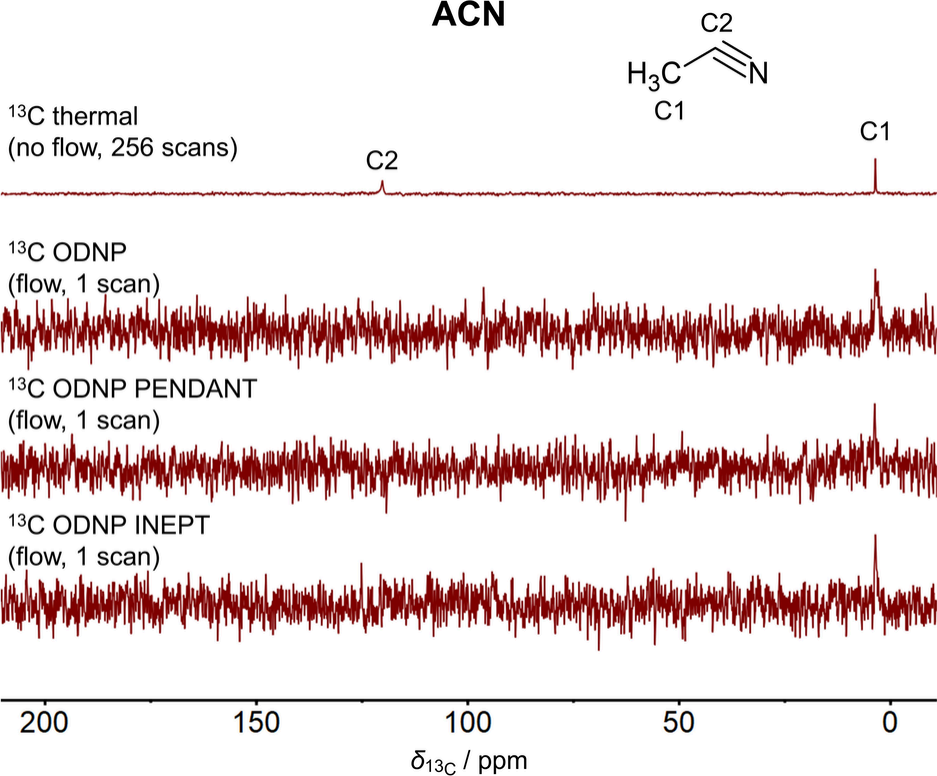
Comparison of ^13^C NMR spectra of ACN acquired by the ^13^C thermal, ^13^C ODNP, ^13^C ODNP PENDANT, and ^13^C ODNP INEPT experiment. The spectrum of the ^13^C thermal experiment was scaled with respect to its number of scans  The experiments with ODNP enhancement were performed at a flow velocity of *v* = 0.85 m s^–1^.

Figure 3 displays the ^13^C NMR spectra of ACN obtained by the ^13^C ODNP, ^13^C ODNP PENDANT, and ^13^C ODNP INEPT experiments in continuous-flow. For comparison, a spectrum of the ^13^C thermal experiment is given. All ODNP-enhanced spectra show the CH_3_-signal (C1) of ACN with single scan acquisition. This is impossible with thermal ^13^C NMR detection due to insufficient premagnetization and SNR as discussed before. Compared to the ^13^C NMR spectrum obtained from the static ^13^C thermal experiment, the C1 peak is broader in all ODNP experiments. This is due to the flow-induced line broadening caused by the reduction of the spin–spin relaxation time *T*_2_. The application of the stop-flow technique would improve the ODNP-enhanced ^13^C NMR spectra by reducing flow effects. This will be investigated in future work.

Moreover, MW-induced heating of the sample has to be considered, which leads to a small line shift of the ACN peak. This is well observed in the ^1^H ODNP experiment. At low flow velocities a larger line shift is observed than at fast flow velocities due to the longer residence time of the fluid in the ODNP probe. The largest line shift was about 13 Hz at a flow velocity of *v* = 0.17 m s^–1^. Furthermore, the heating is mitigated during transport in a 0.25 mm capillary.

**Figure 4:**
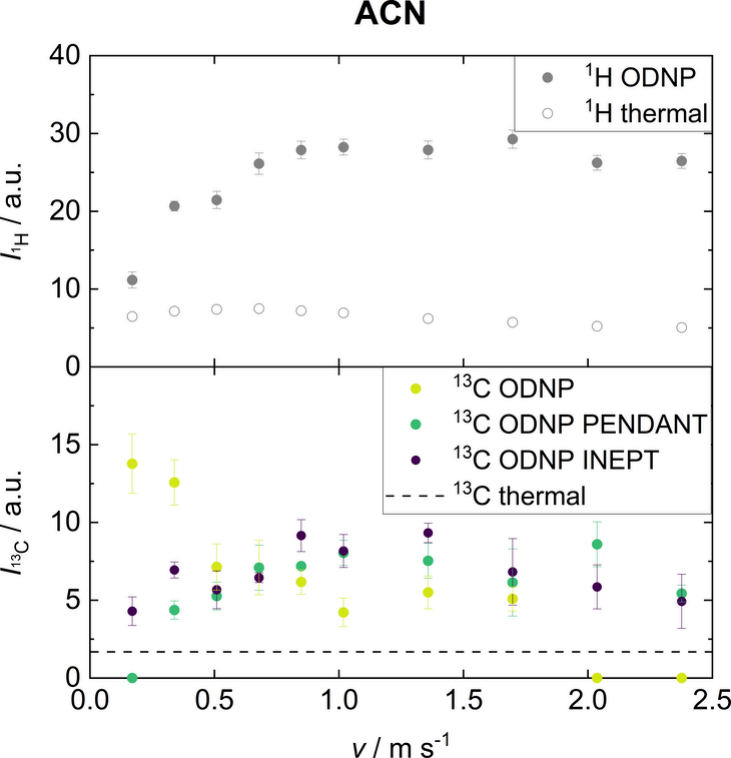
Integrals of signals from ACN (C1) obtained by single scan ^1^H ODNP, ^13^C ODNP, ^13^C ODNP PENDANT, and ^13^C ODNP INEPT at different flow velocities. Also the corresponding integrals obtained in ^1^H and ^13^C thermal experiments, which were obtained with 256 scans, are shown.

An overview of the signal integrals of ACN obtained at different flow velocities is given in Figure 4. The integrals of the ^1^H thermal experiment decrease slightly with increasing flow velocity due to insufficient premagnetization because of the short residence time of the sample in the magnetic field. However, the effect of insufficient premagnetization is not significant because fully premagnetized molecules are present in the detection cell due to the back-mixing effects of the jet-flow. In contrast, the corresponding integral of the ^1^H ODNP experiment first increases with increasing flow velocity and reaches a plateau for values larger than *v* = 1.0 m s^–1^. The initial increase and the plateau are consequences of the reduction of the transport time and therefore of the reduction of the hyperpolarization losses. The maximum signal enhancement is about *E*_^1^H_ = 5.

The largest integral of the ^13^C ODNP experiment is detected at the lowest flow velocity (*v* = 0.17 m s^–1^) and corresponds to a signal enhancement of *E*_^13^C_ = 8. At higher flow velocities, the performance of the ^13^C ODNP experiment decreases significantly, as the time for the interaction with the radical matrix is not sufficient for a complete ODNP hyperpolarization build-up (compare the  value of ACN in Table 3). Therefore, the application of low flow velocities is beneficial for direct ^13^C ODNP. Morerover, since the native  value is much larger than that of ^1^H the small loss of ODNP hyperpolarization during the transport to the benchtop NMR spectrometer is not important here.

In contrast, the ^13^C ODNP PENDANT and ^13^C ODNP INEPT experiments yield larger integrals with increasing flow velocity. Both indirect ODNP methods rely on the hyperpolarization build-up of the ^1^H nuclei, which occurs on a much shorter time scale than for the ^13^C nuclei. The use of high flow rates is, hence, less problematic. As seen before, ^1^H ODNP hyperpolarization losses occur during the transport. Therefore, the application of higher flow velocities preserves the ^1^H ODNP hyperpolarization, which can then be transferred to the ^13^C nuclei. However, a new effect occurs that leads to a decrease in signal at flow velocities above *v* = 1.70 m s^–1^: the application of the polarization transfer sequences takes time. Consequently, a flow rate that is too high results in a less efficient transfer of the ^1^H ODNP hyperpolarization to the ^13^C nuclei. The differences between PENDANT and INEPT results are not significant.

Both approaches, direct ODNP (^13^C ODNP) and indirect ODNP (^13^C ODNP PEN-DANT/^13^C ODNP INEPT), when applied to ACN, have their advantages at different flow regimes: If low flow velocities are applied, direct ODNP is preferable because higher ^13^C signal enhancements can be achieved. The contact time with the radical matrix is sufficient and since the  is long, no significant loss of ODNP hyperpolarization occurs. If high flow velocities are used, indirect ODNP gives better results, as the much shorter  provides a much faster hyperpolarization build-up. However, both approaches share the ability to detect the ACN signal in continuous-flow within a single scan which is impossible without ODNP. Hence, ODNP is an enabling technology for benchtop ^13^C NMR spectroscopy.

### ODNP Experiments with CF

**Figure 5:**
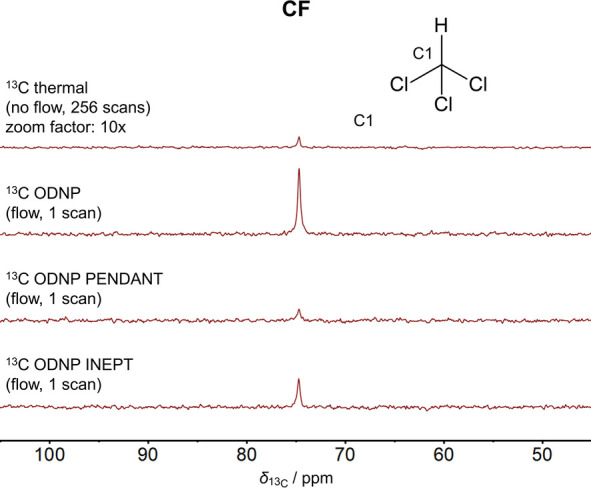
Comparison of ^13^C NMR spectra of CF acquired by the ^13^C thermal, ^13^C ODNP, ^13^C ODNP PENDANT, and ^13^C ODNP INEPT experiment. The spectrum of the ^13^C thermal experiment was scaled with respect to its number of scans . The experiments with ODNP enhancement were performed at a flow velocity of *v* = 1.02 m s^–1^.

Figure 5 illustrates the ^13^C NMR spectra of CF obtained by the ^13^C ODNP, ^13^C ODNP PENDANT, and ^13^C ODNP INEPT experiments in continuous-flow. For comparison, a spectrum of the ^13^C thermal experiment is given. It can be seen that applying ODNP increases the SNR tremendously, although only a single scan was acquired. The direct ODNP shows the best performance.

**Figure 6:**
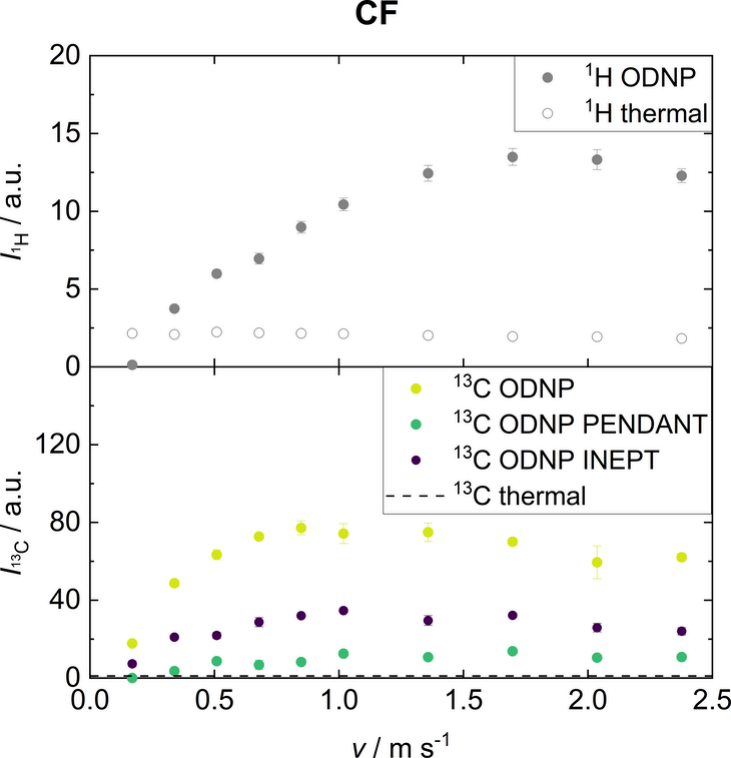
Integrals of signals from CF (C1) obtained by single scan ^1^H ODNP, ^13^C ODNP, ^13^C ODNP PENDANT, and ^13^C ODNP INEPT at different flow velocities. Also the corresponding integrals obtained in ^1^H and ^13^C thermal experiments, which were obtained with 256 scans, are shown.

The integrals of all experiments as a function of the flow velocity are given in Figure 6. The results of the ^1^H thermal as well as the ^1^H ODNP experiments show the same behavior as observed for ACN. However, compared to the ^13^C results of ACN, a significantly larger signal enhancement was observed for CF in all experiments. E.g., for the ^13^C ODNP experiment the largest signal enhancement was about *E*_^13^C_ = 72 compared to a corresponding value of *E*_^13^C_ = 8 for ACN. This observation can be explained by the scalar coupling mechanism, which is strongly dominant for CF due to its molecular structure. A reduction of the net signal enhancement by the contribution of the dipolar coupling and its negative signal is thus prevented. This is in agreement with the results from other groups.^59,62,64,65^ The observed signal enhancements are in a similar range as those found by Dorn et al.^62^ and Stevenson et al.^64^

In contrast to ACN, the largest ^13^C ODNP signal enhancement is not observed for the lowest flow rate for CF - instead, the signal first rises with increasing flow rate, similar to the results for ^1^H ODNP. A reason for the important differences of the results for the direct ^13^C ODNP between ACN and CF may be a difference in the hyperfine interaction of these molecules to the TEMPO radical. For CF, the scalar coupling is largely dominant, whereas for ACN both scalar and dipolar coupling mechanisms should play a role. However, scalar and dipolar coupling lead to signals of different sign that cancel out. The direct ^13^C ODNP experiments of ACN show a small positive signal indicating that both coupling mechanisms are present. Moreover, the strength of the scalar hyperfine interaction is different for both molecules which could also be reflected in different times required for hyperpolarization build-up (usually stronger couplings result in faster polarization transfer). However, a clarification of this issue was out of the scope of the present work, but we plan to investigate this effect in the future work.

For ^13^C ODNP PENDANT and ^13^C ODNP INEPT the observations made for ACN also apply to CF. In general, the indirect ODNP approach results in significantly lower ^13^C signal enhancement than the direct ^13^C ODNP because this method uses the weaker dipolar ^1^H signal enhancement. The signal enhancements obtained by ^13^C ODNP PENDANT are significantly lower than those of ^13^C ODNP INEPT. This is due to the nature of the PENDANT pulse sequence, which allows the simultaneous detection of the original ^13^C polarization and the transferred polarization from ^1^H (this is the reason why quaternary carbons can be detected with PENDANT). In ODNP, the signal enhancements resulting from scalar and dipolar interactions are of opposite sign, thus, reducing the net signal enhancement. In contrast, the refocused INEPT^+^ pulse sequence only permits the detection of ^13^C polarization that originates from transfer from ^1^H.

In summary, ^13^C NMR spectra with very good SNR were obtained with a single scan for CF with a 1 T benchtop NMR spectrometer without using ^13^C enriched substance and in continuous-flow in a flow cell with an inner diameter of only *d* = 2.9 mm. To the best of our knowledge, this observation has not been made before in a comparable setup.

### ODNP Experiments with ACN + CF

**Figure 7:**
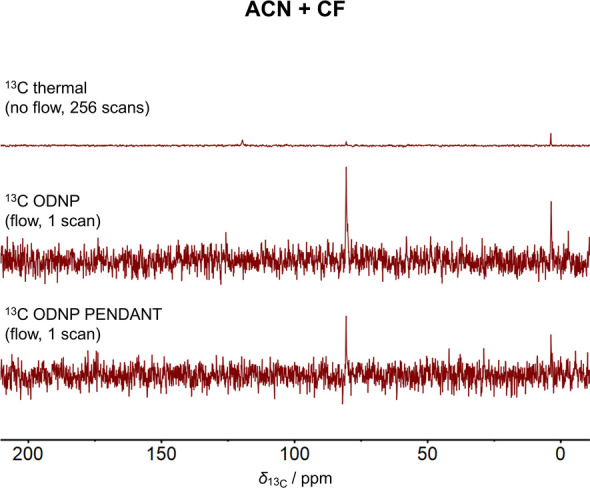
Comparison of ^13^C NMR spectra of the binary mixture ACN and CF (*x*_ACN_ = 0.75 mol mol^–1^) acquired by the ^13^C thermal, ^13^C ODNP, and ^13^C ODNP PENDANT experiment. The spectrum of the ^13^C thermal experiment was scaled with respect to its number of scans  The experiments with ODNP enhancement were performed at a flow velocity of *v* = 0.68 m s^–1^.

To investigate the potential of ODNP on ^13^C for process monitoring, also a binary mixture was studied. ^13^C NMR spectra of the binary mixture consisting of ACN and CF (*x*_ACN_ = 0.75 mol mol^–1^) obtained by ^13^C thermal, ^13^C ODNP, and ^13^C ODNP PENDANT in continuous-flow are shown in Figure 7. ^13^C ODNP INEPT experiments could not be performed due to hardware issues. A significant improvement of the SNR in the ^13^C NMR spectrum by the direct as well as the indirect approach is recognizable for both components.

**Figure 8:**
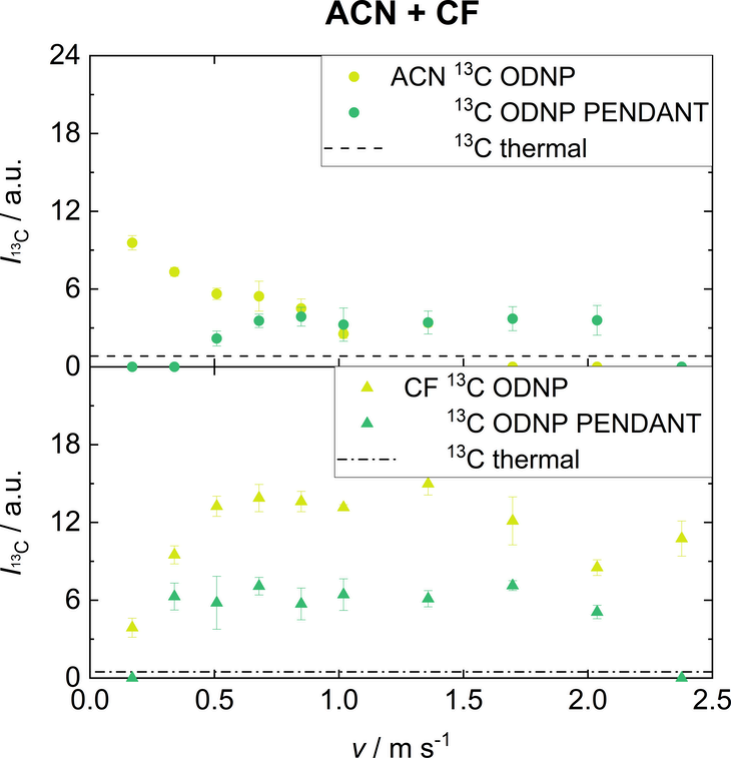
Integrals of signals from ACN + CF (C1 for both molecules) in a binary mixture (*x*_ACN_ = 0.75 mol mol^–1^) obtained by single scan ^13^C ODNP and ^13^C ODNP PENDANT at different flow velocities. Also the corresponding integrals obtained in ^13^C thermal experiments, which were obtained with 256 scans, are shown.

Figure 8 shows the integrals of ACN and CF in the binary mixture obtained from the ^13^C ODNP and the ^13^C ODNP PENDANT experiments as a function of the flow velocity. The observations made for the pure components also apply to the studied binary mixture (compare Figure 4 and Figure 6). Compared to the pure components, lower absolute signal integrals are observed due to the lower total amount of spins of each molecule in the detection cell. However, the signal enhancements obtained in the binary mixture, with the exception of the ^13^C ODNP experiment of CF, are in the same range as for the pure components. The exception for CF can be explained by the fact that in the binary mixture, CF molecules compete with ACN for the limited radical binding sites. Furthermore, it is observed that the ratio between the signal integrals of ACN and CF obtained in the ^13^C ODNP and ^13^C ODNP PENDANT experiments is not the same as that observed in the ^13^C thermal experiments. This is due to differences in the hyperpolarization build-up times and the *T*_1_ relaxation times for the two molecules.

In summary, in the ODNP experiments the detection of the ^13^C signals of both components is possible even in continuous-flow. This is not the case for ^13^C thermal experiments. The application of ^13^C ODNP hyperpolarization therefore opens the route for quantifying the composition of flowing mixtures by ^13^C NMR, e.g., in reaction kinetic studies. The present results suggest that this is possible, but requires a calibration. In preliminary studies, a suitable flow rate should be chosen, for which at least one signal for each component is detectable. Then, the calibration can be carried out with mixtures of known composition. However, in a recent publication by van der Ham,^81^ an approach was proposed that could overcome a possible calibration procedure.

## Conclusions

In this work, we have applied ODNP hyperpolarization to continuous-flow benchtop ^13^C NMR spectroscopy. To the best of our knowledge, this is the first study of this technique, which is highly attractive for reaction and process monitoring. Three ODNP methods were studied: direct ODNP (^13^C ODNP) and two indirect ^1^H–^13^C ODNP methods (^13^C ODNP PENDANT and ^13^C ODNP INEPT). Their performance was evaluated at different flow velocities for pure acetonitrile (ACN), chloroform (CF), methanol (MeOH), and a mixture of acetonitrile and chloroform (ACN + CF). Significant ^13^C signal enhancements were found in basically all cases. Even though no ^13^C enriched substances were used, it was shown that single scan ODNP experiments can yield ^13^C NMR spectra with good SNR even at high flow velocities. The size of the signal enhancement and its dependency on the flow velocity is different for the different studied ODNP techniques and it also depends on the investigated substance. The actual outcome is determined by several effects, starting with the efficiency of the polarization transfer in the fixed bed (to the ^13^C nuclei in the direct method and to the ^1^H nuclei in the indirect method) and the loss of polarization on the way from the fixed bed to the NMR detection volume. These two effects depend on the corresponding *T*_1_ times which are much lower for ^1^H than for ^13^C. Furthermore, effects in the detection volume are important, where in the indirect methods the transfer of the polarization from ^1^H to ^13^C has to be accomplished as well as in all cases the final ^13^C NMR experiment must be carried out. The influence of all these effects on the results of the different ODNP methods was elucidated and discussed for the different studied substances and conditions. Besides these results of studies on ^13^C ODNP, also results from corresponding ^1^H NMR measurements are reported, in which also important enhancements were observed.

The preset studies should be extended in several directions: the design of the NMR detection flow cell should be improved. The jump of the diameter at the inlet of the present cell is certainly not an optimal design, a smooth transition, that avoids back-mixing and dead zones will enable much higher enhancements than those obtained in the present work. Furthermore, a combination with ultrafast (UF)-2D NMR^82−85^ techniques could be explored.

The present work lays the foundation for the application of benchtop ^13^C NMR spectroscopy in flow for the quantification of mixtures. This would open new perspectives for reaction and process monitoring.

## Supporting Information Available

Additional experimental details, numerical values, and signal enhancements (PDF)
