# Supplementary material for: Overhauser Dynamic Nuclear Polarization Enables Single Scan Benchtop 13C NMR Spectroscopy in Continuous-Flow
Source: Anal Chem. 2025 Feb 21;97(8):4308–17. doi: 10.1021/acs.analchem.4c03985 (PMC11883742; doi:10.1021/acs.analchem.4c03985)
Supplement: Supplementary file 1 — ac4c03985_si_001.pdf [file ac4c03985_si_001.pdf]

Supporting Information
Overhauser Dynamic Nuclear Polarization Enables Single Scan Benchtop ^{13}C NMR Spectroscopy in Flow

Johnnie Phuong,[a,b] Billy Salgado,[a,b] Tom Labusch,[a,b] Hans Hasse,[a,b] and Kerstin Münnemann*,[a,b]

[a]*Laboratory of Engineering Thermodynamics (LTD), RPTU Kaiserslautern, Erwin-Schrödinger-Straße 44, 67663 Kaiserslautern, Germany*

[b]*Laboratory of Advanced Spin Engineering - Magnetic Resonance (LASE-MR), RPTU Kaiserslautern, Gottlieb-Daimler-Straße 76, 67663 Kaiserslautern, Germany*

* E-mail: *kerstin.muennemann@rptu.de

Contents

SI.1 Experimental Section **S3**

SI.2 Results and Discussion **S5**

SI.2.1 Characterization of the Flow Cell S5

SI.2.2 ODNP Experiments with ACN S6

SI.2.3 ODNP Experiments with CF S10

SI.2.4 ODNP Experiments with ACN + CF S13

SI.2.5 ODNP Experiments with MeOH S18

SI.1 Experimental Section

Figure SI.1 illustrates the used pulse sequence PENDANT for the polarization transfer from ^1H to the ^{13}C nuclei. The applied evolution delays are given in Equation (SI.1) and (SI.2).

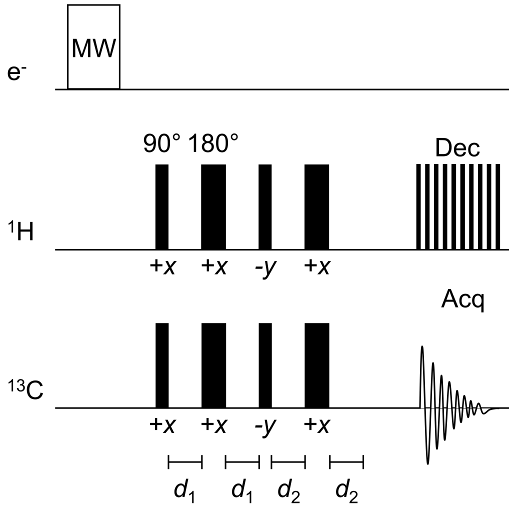

Figure SI.1: Illustration of the used polarization transfer pulse sequence PENDANT.

$$d_1 = \frac{1}{4^1 J_{\mathrm{C,H}}} \tag{SI.1}$$

$$d_2 = \frac{5}{8^1 J_{\mathrm{C,H}}} \tag{SI.2}$$

Figure SI.2 displays the pulse sequence refocused INEPT$^+$ also for the polarization transfer from ^1H to the ^{13}C nuclei. The applied evolution delays are given in Equation (SI.3) and (SI.4).

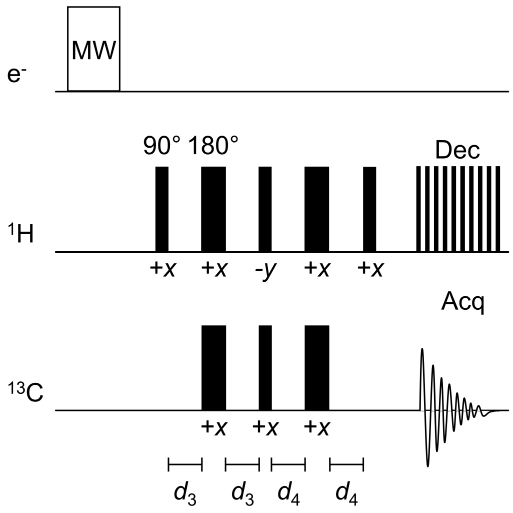

Figure SI.2: Illustration of the used polarization transfer pulse sequence INEPT.

$$d_3 = \frac{1}{4^1 J_{\mathrm{C,H}}} \tag{SI.3}$$

$$d_4 = \frac{1}{6^1 J_{\mathrm{C,H}}} \tag{SI.4}$$

The error as well as the error bars of the signal enhancements are calculated with an error propagation which is given in Equation SI.5. For the estimation of ΔI_{ODNP} and $\Delta I_{\mathrm{thermal}}$ the specific standard uncertainty of three experiments is applied.

$$\Delta E = \left| \left(\frac{\sqrt{n_{\mathrm{thermal}}}}{\sqrt{n_{\mathrm{ODNP}}}} \cdot \frac{1}{I_{\mathrm{thermal,\,scaled}}} \right) \right| \cdot \Delta I_{\mathrm{ODNP}} + \left| \left(- \frac{\sqrt{n_{\mathrm{thermal}}}}{\sqrt{n_{\mathrm{ODNP}}}} \cdot \frac{I_{\mathrm{ODNP}}}{I^2_{\mathrm{thermal,\,scaled}}} \right) \right| \cdot \Delta I_{\mathrm{thermal}} \tag{SI.5}$$

SI.2 Results and Discussion

SI.2.1 Characterization of the Flow Cell

In a step experiment in which a fluid in the detection cell was replaced by another the fluid dynamic within the flow cell was characterized. For this study, the flow cell, which was exactly positioned in the benchtop NMR spectrometer as in the later ODNP experiments, was filled with ACN in the first step. ACN was replaced by water (W) after activating the pump and, hence, the flow ($\dot{V}$ = 2.0 ml min^{-1} corresponding to v = 0.68 m s^{-1}). The change of the composition in the flow cell was monitored by ^{1}H NMR experiments (1 scan every 5 s, 90° excitation pulse, 1.6 s acquisition time, 8192 data points) for 400 s. The experiment was performed three times. Figure SI.3 illustrates the result of the tracer experiment and displays the relative signal change of W (S_{W}) in dependence of the relative time (Θ). The definition for Θ is given in Equation SI.6. Here, t denotes the experimental time and t_{total} the total experimental time of the step experiment, which was $t_{\mathrm{total}} = 395$ s.

The signal of W increases during the time as W is replacing ACN in the flow cell. A large deviation from an ideal flow cell without any back-mixing effect (ACN is instantly replaced by W) is observed. This results in a detection of a mixture of ODNP hyperpolarized and thermally polarized molecules which underestimates the calculated signal enhancements.

$$\Theta = \frac{t}{t_{\mathrm{total}}} \tag{SI.6}$$

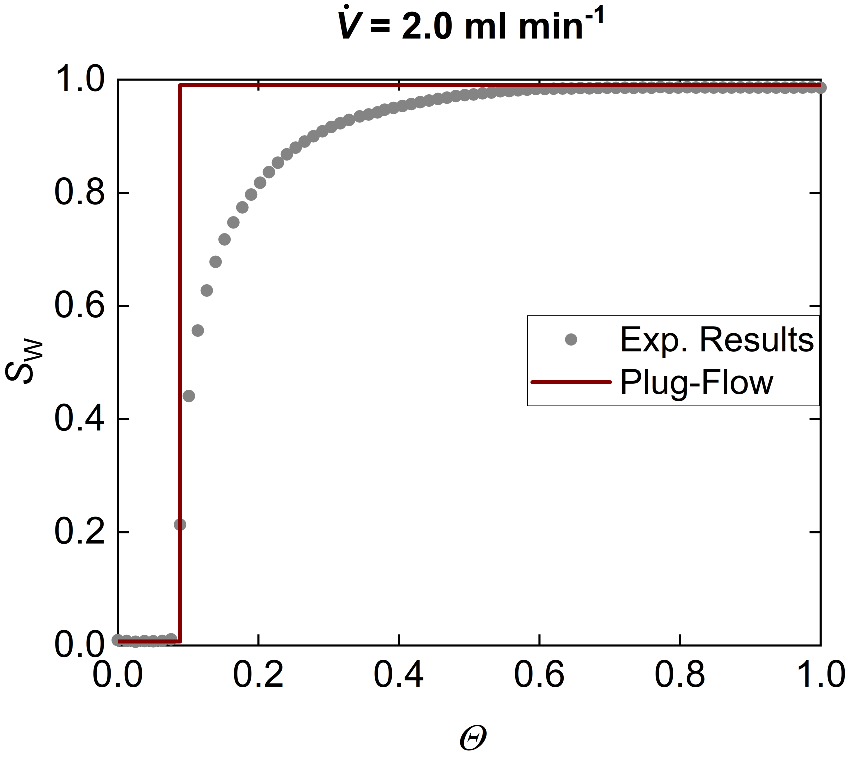

Figure SI.3: The cell was initially filled with ACN which was then replaced by W for the fluid dynamic characterisation of the flow cell at a flow rate of $\dot{V} = 2.0$ ml min^{-1} (corresponding to a flow velocity of $v = 0.68$ m s^{-1}). The relative signal of W (S_{W}) is obtained by ^{1}H NMR experiments in dependence of the relative time Θ. For comparison the signal of an ideal plug-flow is given.

SI.2.2 ODNP Experiments with ACN

Table SI.1 displays the signal integrals and the signal enhancements of ACN acquired with ^{1}H ODNP, ^{13}C ODNP, ^{13}C ODNP PENDANT, and ^{13}C ODNP INEPT at different flow velocities. Note that the integral of the Boltzmann thermal equilibrium experiment, which is used for the calculation of the signal enhancement of all ^{13}C ODNP experiments, was obtained without any flow and with 256 scans. The signal enhancements are additionally visualized in Figure SI.4.

Table SI.1: Numerical results of the ODNP experiments with ACN (C1) at different flow velocities. The scaled integral of the Boltzmann thermal equilibrium experiment ($I_{\text{thermal, scaled}}$) as well as of the signals of the ODNP experiments ^{1}H ODNP, ^{13}C ODNP, ^{13}C ODNP PENDANT, and ^{13}C ODNP INEPT ($I_{\text{ODNP, scaled}}$) are given. Errors are calculated from three repetitions using standard uncertainty. The signal enhancement E is provided as well as the result of the error propagation.

	^{1}H ODNP			^{13}C ODNP		
v / m s^{-1}	$I_{\text{thermal, scaled}}$ / a.u.	$I_{\text{ODNP, scaled}}$ / a.u.	E	$I_{\text{thermal, scaled}}$ / a.u.	$I_{\text{ODNP, scaled}}$ / a.u.	E
0.17	6.5±0.1	11.2±1.0	1.7±0.2	1.7±0.1	13.8±1.9	8.2±1.5
0.34	7.1±0.1	20.7±0.6	2.9±0.1		12.6±1.5	7.5±1.2
0.51	7.4±0.1	21.4±1.1	2.9±0.2		7.1±1.5	4.2±1.1
0.68	7.5±0.1	26.1±1.4	3.5±0.2		7.1±1.8	4.2±1.2
0.85	7.2±0.1	27.9±1.1	3.9±0.2		6.2±0.8	3.7±0.6
1.02	6.9±0.1	28.2±1.0	4.1±0.2		4.2±0.9	2.5±0.6
1.36	6.2±0.1	27.9±1.1	4.5±0.2		5.5±1.1	3.3±0.8
1.70	5.7±0.1	29.3±1.2	5.1±0.2		5.1±0.8	3.0±0.6
2.04	5.2±0.1	26.2±0.9	5.0±0.2		0	0
2.38	5.0±0.1	26.5±0.9	5.2±0.2		0	0

v / m s^{-1}	^{13}C ODNP PENDANT $I_{\text{thermal, scaled}}$ / a.u.	$I_{\text{ODNP, scaled}}$ / a.u.	E	^{13}C ODNP INEPT $I_{\text{thermal, scaled}}$ / a.u.	$I_{\text{ODNP, scaled}}$ / a.u.	E
0.17	1.7±0.1	0	0	1.7±0.1	4.3±0.9	2.6±0.7
0.34		4.4±0.6	2.6±0.5		6.9±0.5	4.1±0.5
0.51		5.3±0.9	3.1±0.7		5.7±1.2	3.4±0.9
0.68		7.1±1.5	4.2±1.0		6.4±0.3	3.8±0.3
0.85		7.2±0.1	4.3±0.3		9.2±1.0	5.4±0.8
1.02		8.0±0.8	4.8±0.7		8.2±1.1	4.9±0.8
1.36		7.5±1.1	4.5±0.8		9.3±0.6	5.6±0.6
1.70		6.1±2.2	3.7±1.4		6.8±2.1	4.1±1.4
2.04		8.6±1.4	5.1±1.1		5.9±1.4	3.5±1.0
2.38		5.4±0.5	3.2±0.5		4.9±1.7	2.9±1.2

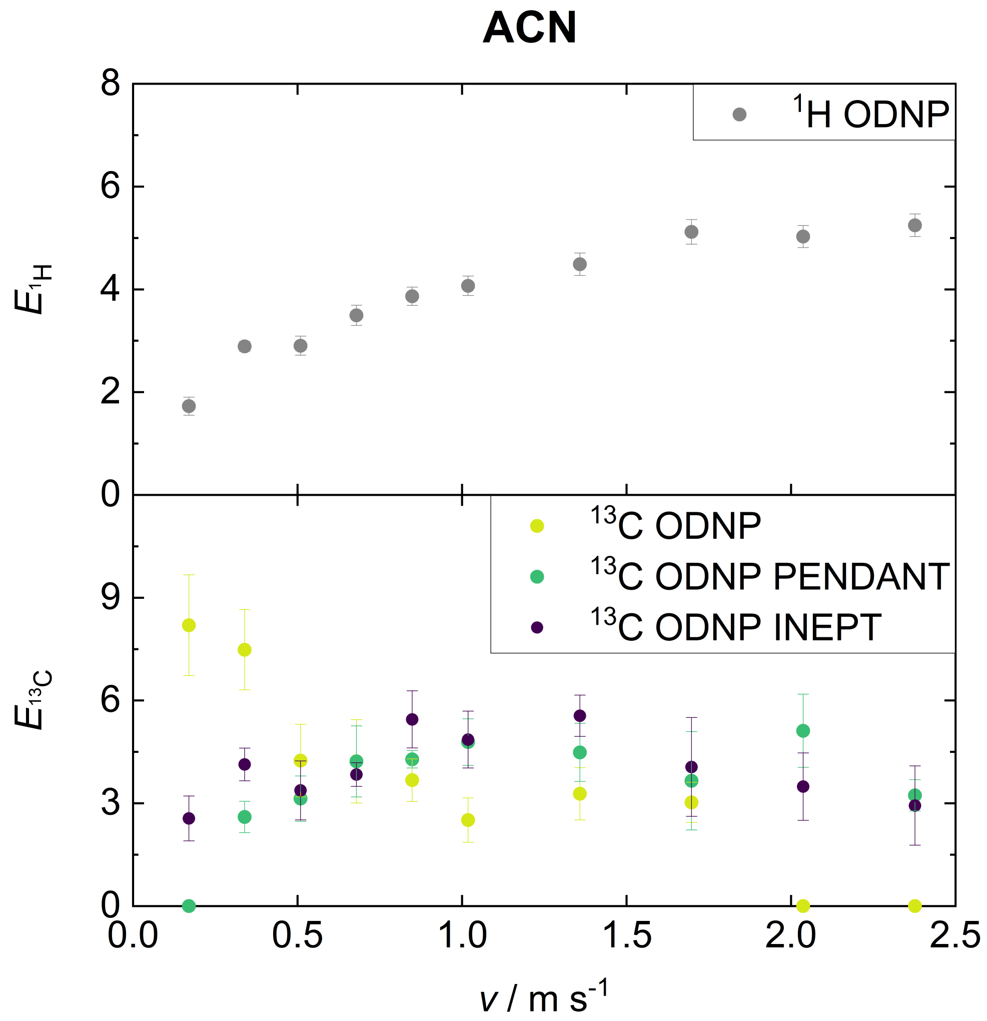

Figure SI.4: Signal enhancements of ACN (C1) obtained by ^{1}H ODNP, ^{13}C ODNP, ^{13}C ODNP PENDANT, and ^{13}C ODNP INEPT at different flow velocities.

SI.2.3 ODNP Experiments with CF

Table SI.2 displays the signal integrals and the signal enhancements of CF acquired with ^{1}H ODNP, ^{13}C ODNP, ^{13}C ODNP PENDANT, and ^{13}C ODNP INEPT at different flow velocities. Note that the integral of the Boltzmann thermal equilibrium experiment, which is used for the calculation of the signal enhancement of all ^{13}C ODNP experiments, was obtained without any flow and with 256 scans. The signal enhancements are additionally visualized in Figure SI.5.

Table SI.2: Numerical results of the ODNP experiments with CF (C1) at different flow velocities. The scaled integral of the Boltzmann thermal equilibrium experiment ($I_{\text{thermal, scaled}}$) as well as of the signals of the ODNP experiments ^{1}H ODNP, ^{13}C ODNP, ^{13}C ODNP PENDANT, and ^{13}C ODNP INEPT ($I_{\text{ODNP, scaled}}$) are given. Errors are calculated from three repetitions using standard uncertainty. The signal enhancement E is provided as well as the result of the error propagation.

	^{1}H ODNP			^{13}C ODNP		
v / m s^{-1}	$I_{\text{thermal, scaled}}$ / a.u.	$I_{\text{ODNP, scaled}}$ / a.u.	E	$I_{\text{thermal, scaled}}$ / a.u.	$I_{\text{ODNP, scaled}}$ / a.u.	E
0.17	2.1±0.1	0.1±0.1	0.1±0.1	1.1±0.1	17.8±1.2	16.5±1.6
0.34	2.1±0.1	3.7±0.2	1.8±0.1		48.7±1.7	45.1±2.8
0.51	2.2±0.1	6.0±0.2	2.7±0.1		63.4±2.5	58.7±3.9
0.68	2.2±0.1	7.0±0.3	3.2±0.2		72.7±0.9	67.3±2.6
0.85	2.1±0.1	9.0±0.4	4.2±0.2		77.2±3.6	71.5±5.2
1.02	2.1±0.1	10.4±0.4	4.9±0.3		74.2±5.1	68.7±6.5
1.36	2.0±0.1	12.4±0.5	6.2±0.3		74.9±4.7	69.4±6.2
1.70	1.9±0.1	13.5±0.5	7.0±0.3		70.1±1.8	64.9±3.3
2.04	1.9±0.1	13.1±0.6	6.9±0.4		59.4±8.4	55.0±9.3
2.38	1.8±0.1	12.3±0.4	6.7±0.3		62.1±2.1	57.5±3.5

v / m s^{-1}	^{13}C ODNP PENDANT $I_{\text{thermal, scaled}}$ / a.u.	$I_{\text{ODNP, scaled}}$ / a.u.	E	^{13}C ODNP INEPT $I_{\text{thermal, scaled}}$ / a.u.	$I_{\text{ODNP, scaled}}$ / a.u.	E
0.17	1.1±0.1	0	0	1.1±0.1	7.3±1.4	6.8±1.5
0.34		3.6±0.2	3.3±0.3		21.0±1.2	19.5±1.6
0.51		8.7±1.7	8.0±1.8		21.9±1.6	20.3±2.0
0.68		6.7±2.4	6.2±2.3		28.7±2.4	26.6±2.9
0.85		8.3±0.5	7.6±0.6		32.0±0.3	29.6±1.1
1.02		12.5±1.4	11.6±1.6		34.7±0.7	32.1±1.5
1.36		10.7±0.5	9.9±0.7		29.6±2.5	27.4±3.0
1.70		13.8±0.6	12.7±0.9		32.2±1.2	29.8±1.9
2.04		10.4±0.6	9.7±0.8		25.9±2.2	24.0±2.7
2.38		10.8±1.2	10.0±1.3		24.0±1.9	22.3±2.4

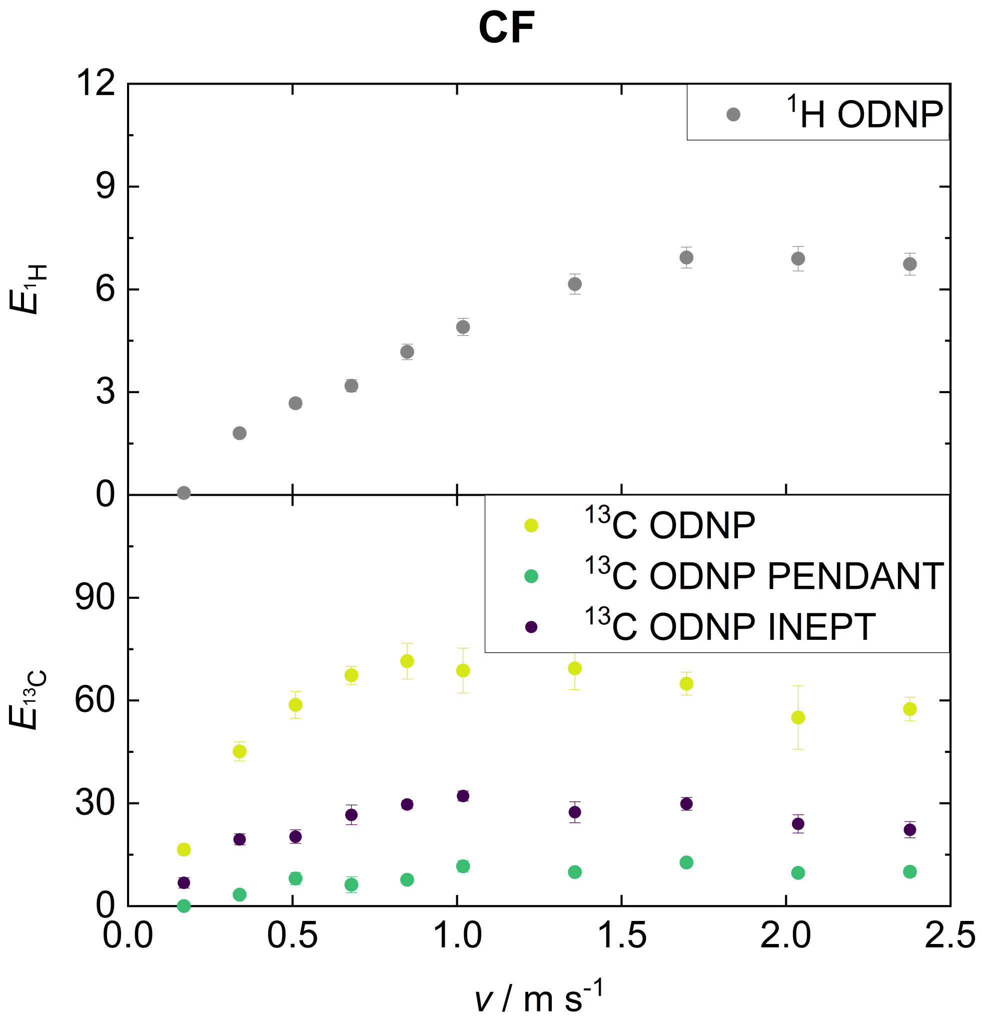

Figure SI.5: Signal enhancements of CF (C1) obtained by ^{1}H ODNP, ^{13}C ODNP, ^{13}C ODNP PENDANT, and ^{13}C ODNP INEPT at different flow velocities.

SI.2.4 ODNP Experiments with ACN + CF

Table SI.3 displays the signal integrals and the signal enhancements of ACN and CF in the binary mixture ($x_{\mathrm{ACN}} = 0.75$ mol mol^{-1}) acquired with ^{1}H ODNP, ^{13}C ODNP, and ^{13}C ODNP PENDANT at different flow velocities. Note that the integral of the Boltzmann thermal equilibrium experiment, which is used for the calculation of the signal enhancement of all ^{13}C ODNP experiments, was obtained without any flow and with 256 scans. The signal enhancements are additionally visualized in Figure SI.6.

Table SI.3: Numerical results of the ODNP experiments with the binary mixture of ACN and CF (x_{ACN} = 0.75 mol mol^{-1}, C1 for both molecules) at different flow velocities. The scaled signals of the integral of the Boltzmann thermal equilibrium experiment ($I_{\mathrm{thermal,\,scaled}}$) as well as of the ODNP experiments ^{1}H ODNP, ^{13}C ODNP, and ^{13}C ODNP PENDANT ($I_{\mathrm{ODNP,\,scaled}}$) are given. Errors are calculated from three repetitions using standard uncertainty. The signal enhancement E is provided as well as the result of the error propagation.

ACN						
	^{1}H ODNP			^{13}C ODNP		
v / m s^{-1}	$I_{\mathrm{thermal,\,scaled}}$ / a.u.	$I_{\mathrm{ODNP,\,scaled}}$ / a.u.	E	$I_{\mathrm{thermal,\,scaled}}$ / a.u.	$I_{\mathrm{ODNP,\,scaled}}$ / a.u.	E
0.17	4.4±0.1	3.2±0.1	0.7±0.1	0.8±0.1	9.6±0.6	11.5±0.9
0.34	4.6±0.1	8.1±0.1	1.8±0.1		7.3±0.3	8.8±0.6
0.51	4.4±0.1	9.3±0.1	2.1±0.1		5.6±0.4	6.8±0.7
0.68	4.5±0.1	12.0±0.1	2.7±0.1		5.4±1.1	6.5±1.5
0.85	4.7±0.1	14.4±0.1	3.0±0.1		4.5±0.7	5.4±1.0
1.02	4.3±0.1	13.8±0.1	3.2±0.1		2.5±0.3	3.1±0.5
1.36	4.2±0.1	13.9±0.1	3.3±0.1		3.4±0.3	4.1±0.5
1.70	3.8±0.1	14.0±0.1	3.6±0.1		0	0
2.04	3.5±0.1	13.9±0.1	4.0±0.1		0	0
2.38	3.4±0.1	13.4±0.1	3.9±0.1		0	0

ACN			
	^{13}C ODNP PENDANT		
v / m s^{-1}	$I_{\text{thermal, scaled}}$ / a.u.	$I_{\text{ODNP, scaled}}$ / a.u.	E
0.17	0.8±0.1	0	0
0.34		0	0
0.51		2.2±0.6	2.6±0.7
0.68		3.6±0.5	4.3±0.7
0.85		3.9±0.7	4.6±1.0
1.02		3.3±1.3	3.9±1.6
1.36		3.4±0.9	4.1±1.1
1.70		3.7±0.9	4.5±1.2
2.04		3.6±1.1	4.3±1.5
2.38		0	0

CF						
	^{1}H ODNP			^{13}C ODNP		
v / m s^{-1}	$I_{\text{thermal, scaled}}$ / a.u.	$I_{\text{ODNP, scaled}}$ / a.u.	E	$I_{\text{thermal, scaled}}$ / a.u.	$I_{\text{ODNP, scaled}}$ / a.u.	E
0.17	0.4±0.1	0.4±0.1	1.2±0.2	0.5±0.1	3.8±0.7	8.2±1.7
0.34	0.4±0.1	0.9±0.1	2.1±0.1		9.4±0.7	20.0±1.9
0.51	0.4±0.1	1.0±0.1	2.4±0.2		13.2±0.8	27.9±2.2
0.68	0.4±0.1	1.3±0.1	3.1±0.2		13.9±1.1	29.2±2.8
0.85	0.5±0.1	1.8±0.1	3.5±0.2		13.6±0.8	28.7±2.2
1.02	0.5±0.1	1.7±0.1	3.8±0.3		13.2±0.2	27.7±1.0
1.36	0.5±0.1	2.0±0.1	4.0±0.3		15.0±0.9	31.5±2.5
1.70	0.4±0.1	1.9±0.1	4.4±0.3		12.1±1.9	25.5±4.4
2.04	0.5±0.1	2.3±0.2	4.7±0.3		8.5±0.6	17.9±1.6
2.38	0.4±0.1	1.7±0.1	4.8±0.4		10.7±1.4	22.6±3.3

CF			
	^{13}C ODNP PENDANT		
v / m s^{-1}	$I_{\text{thermal, scaled}}$ / a.u.	$I_{\text{ODNP, scaled}}$ / a.u.	E
0.17	0.5±0.1	0	0
0.34		6.3±1.0	13.2±2.4
0.51		5.8±2.0	12.2±4.5
0.68		7.1±0.7	14.9±1.7
0.85		5.7±1.2	12.0±2.8
1.02		6.4±1.2	13.5±2.8
1.36		6.1±0.6	12.9±1.6
1.70		7.1±0.4	15.0±1.1
2.04		5.1±0.5	10.7±1.3
2.38		0	0

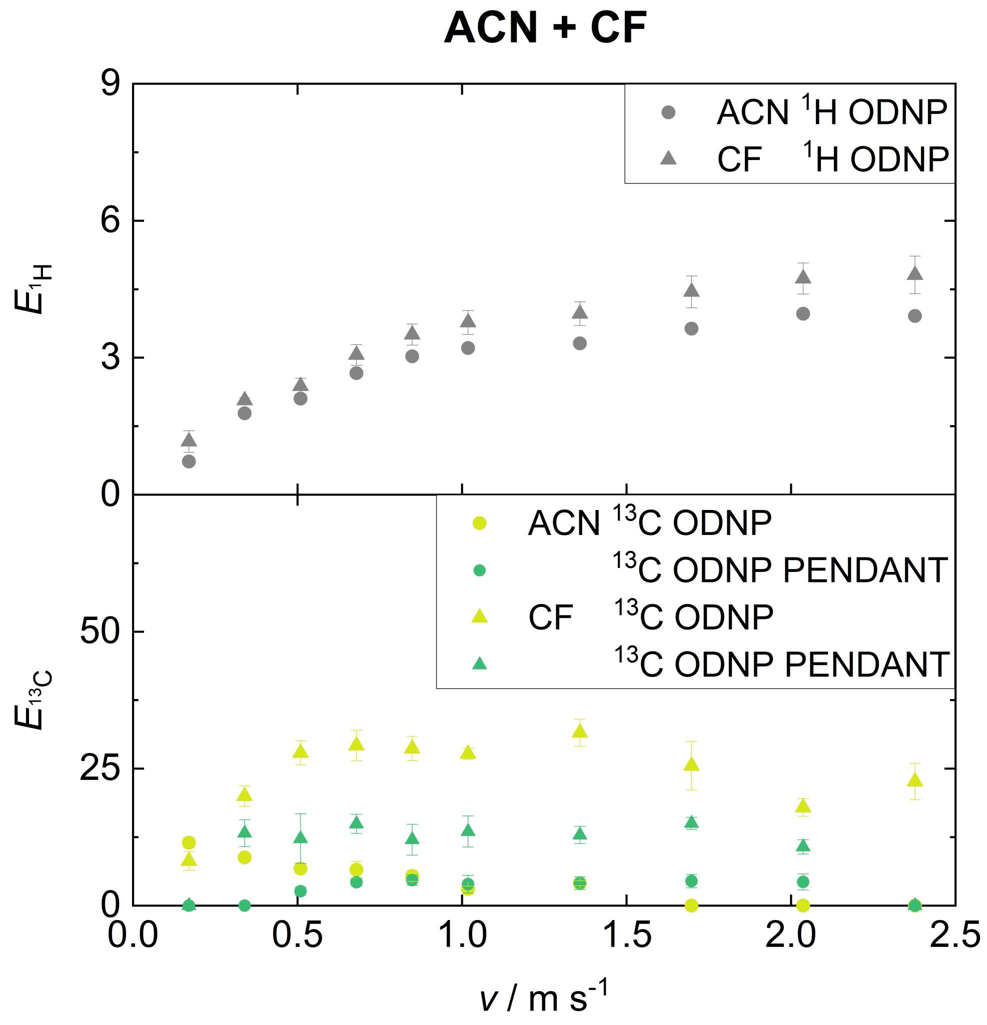

Figure SI.6: Signal enhancements of ACN and CF (C1 for both molecules) in a binary mixture (x_{ACN} = 0.75 mol mol^{-1}) obtained by ^{1}H ODNP, ^{13}C ODNP, and ^{13}C ODNP PENDANT at different flow velocities.

SI.2.5 ODNP Experiments with MeOH

In Table SI.4, the $T_{1,^1H}$ and $T_{1,^{13}C}$ values of MeOH without and with contact to the radical matrix are reported. ^{13}C NMR spectra of MeOH obtained by ^{13}C thermal, ^{13}C ODNP, and ^{13}C ODNP PENDANT in continuous-flow are given in Figure SI.7. ^{13}C ODNP INEPT experiments were not conducted due to failure of the MW amplifier. We expect the results of these experiments to be similar to those of the ACN experiments.

Table SI.4: $T_{1,^1H}$ and $T_{1,^{13}C}$ times values of MeOH without ($T_{1,i}^0$) and with contact to the radical matrix ($T_{1,i}^{RM}$) at $B_0 = 1$ T. Mean value and standard uncertainty from three identical experiments are reported. The $T_{1,^{13}C}$ and $T_{1,^1H}$ values refer to the CH_3-group of MeOH.

	^{1}H		^{13}C	
Molecule	$T_{1,^1H}^0$ / s	$T_{1,^1H}^{RM}$ / s	$T_{1,^{13}C}^0$ / s	$T_{1,^{13}C}^{RM}$ / s
MeOH	2.96 ± 0.04	0.11 ± 0.01	14.05 ± 0.46	0.80 ± 0.01

The integrals as a function of the flow velocity are shown in Figure SI.8. For the ^{1}H thermal and ^{1}H ODNP experiments, comparable observations were made for MeOH as already noticed for ACN and CF. However, it can be seen that the direct ODNP approach does not result in detectable ^{13}C polarization at any flow velocity. In contrast, with ^{13}C ODNP PENDANT a signal can be acquired, but with only moderate enhancement.

In comparison to ACN and CF, a much lower signal enhancement was obtained on the ^{1}H (max $E_{^1H} = 3$) as well as on the ^{13}C nucleus (max $E_{^{13}C} = 3$, for ^{13}C ODNP PENDANT). The low enhancements can be explained by the poor hyperfine interaction of the CH_3-group of MeOH with the TEMPO radical. The polar molecular structure of the radical interacts preferentially with the equally polar OH-group of MeOH, resulting in a low ODNP hyperpolarization of the ^{1}H and ^{13}C nuclei of the CH_3-group. This is especially critical for the direct ^{13}C ODNP technique, since the weak ^{13}C ODNP hyperpolarization can be additionally cancelled by the competing dipolar (negative signal) and scalar coupling (positive signal). However, indirect ODNP still allows MeOH to be detected by ^{13}C NMR spectroscopy in a single scan in continuous-flow mode because it relies entirely on the available ^{1}H ODNP hyperpolarization. This highlights that there are situations where indirect ODNP can be used when direct ODNP fails.

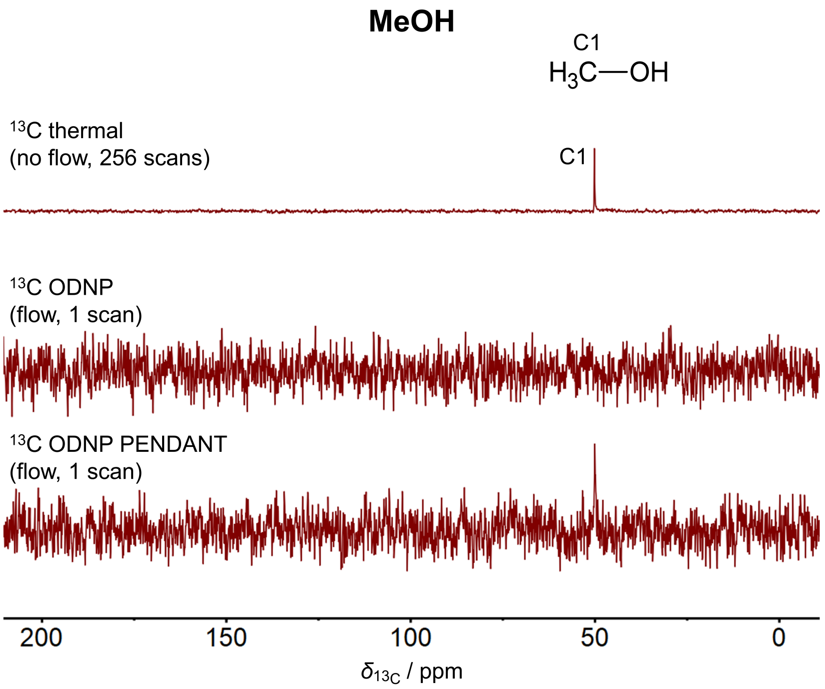

Figure SI.7: Comparison of ^{13}C NMR spectra of MeOH acquired by the ^{13}C thermal, ^{13}C ODNP, and ^{13}C ODNP PENDANT experiment. The spectrum of the ^{13}C thermal experiment was scaled with respect to its number of scans ($\sqrt{n}$). The experiments with ODNP enhancement were performed at a flow velocity of $v = 2.38$ m s^{-1}.

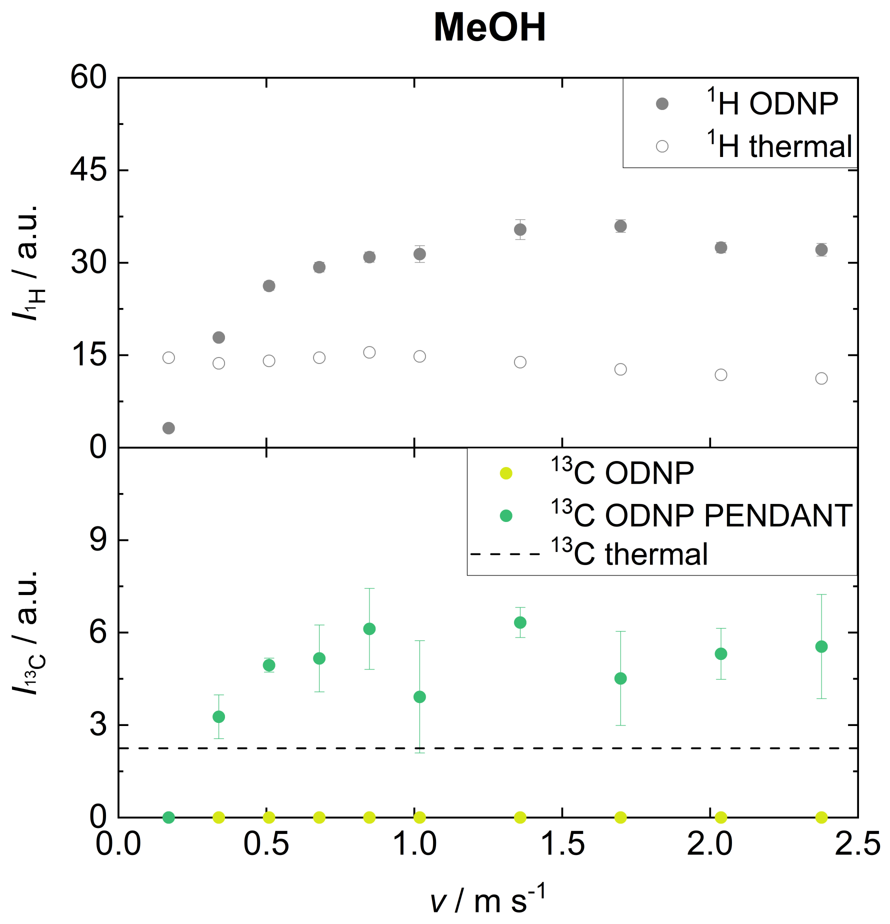

Figure SI.8: Integrals of signals from MeOH (C1) obtained by single scan ^{1}H ODNP, ^{13}C ODNP, and ^{13}C ODNP PENDANT at different flow velocities. Also the corresponding integrals obtained in ^{1}H and ^{13}C thermal experiments, that were obtained with 256 scans, are shown.

Table SI.5 displays the signal integrals and the signal enhancements of MeOH acquired with ^{1}H ODNP, ^{13}C ODNP, and ^{13}C ODNP PENDANT at different flow velocities. Note that the integral of the Boltzmann thermal equilibrium experiment, which is used for the calculation of the signal enhancement of all ^{13}C ODNP experiments, was obtained without any flow and with 256 scans. The signal enhancements are additionally visualized in Figure SI.9.

Table SI.5: Numerical results of the ODNP experiments with MeOH (C1) at different flow velocities. The scaled integral of the Boltzmann thermal equilibrium experiment ($I_{\text{thermal, scaled}}$) as well as of the signals of the ODNP experiments ^{1}H ODNP, ^{13}C ODNP, and ^{13}C ODNP PENDANT ($I_{\text{ODNP, scaled}}$) are given. Errors are calculated from three repetitions using standard uncertainty. The signal enhancement E is provided as well as the result of the error propagation.

	^{1}H ODNP			^{13}C ODNP		
v / m s^{-1}	$I_{\text{thermal, scaled}}$ / a.u.	$I_{\text{ODNP, scaled}}$ / a.u.	E	$I_{\text{thermal, scaled}}$ / a.u.	$I_{\text{ODNP, scaled}}$ / a.u.	E
0.17	14.6±0.3	3.2±0.3	0.2±0.1	2.2±0.1	0	0
0.34	13.7±0.3	17.9±0.5	1.3±0.1		0	0
0.51	14.1±0.2	26.2±0.7	1.9±0.1		0	0
0.68	14.6±0.1	29.3±0.8	2.0±0.1		0	0
0.85	15.4±0.1	30.9±0.8	2.0±0.1		0	0
1.02	14.8±0.1	31.4±1.4	2.1±0.1		0	0
1.36	13.9±0.2	35.4±1.6	2.6±0.1		0	0
1.70	12.7±0.1	35.9±1.0	2.8±0.1		0	0
2.04	11.8±0.1	32.4±0.8	2.8±0.1		0	0
2.38	11.2±0.1	32.1±1.0	2.9±0.1		0	0

v / m s^{-1}	^{13}C DNP PENDANT $I_{\text{thermal, scaled}}$ / a.u.	$I_{\text{ODNP, scaled}}$ / a.u.	E
0.17	2.2±0.1	0	0
0.34		3.3±0.7	1.5±0.4
0.51		4.9±0.2	2.2±0.2
0.68		5.2±1.1	2.3±0.5
0.85		6.1±1.3	2.7±0.7
1.02		3.9±1.8	1.7±0.9
1.36		6.3±0.5	2.8±0.3
1.70		4.5±1.5	2.0±0.7
2.04		5.3±0.8	2.4±0.4
2.38		5.5±1.7	2.5±0.8

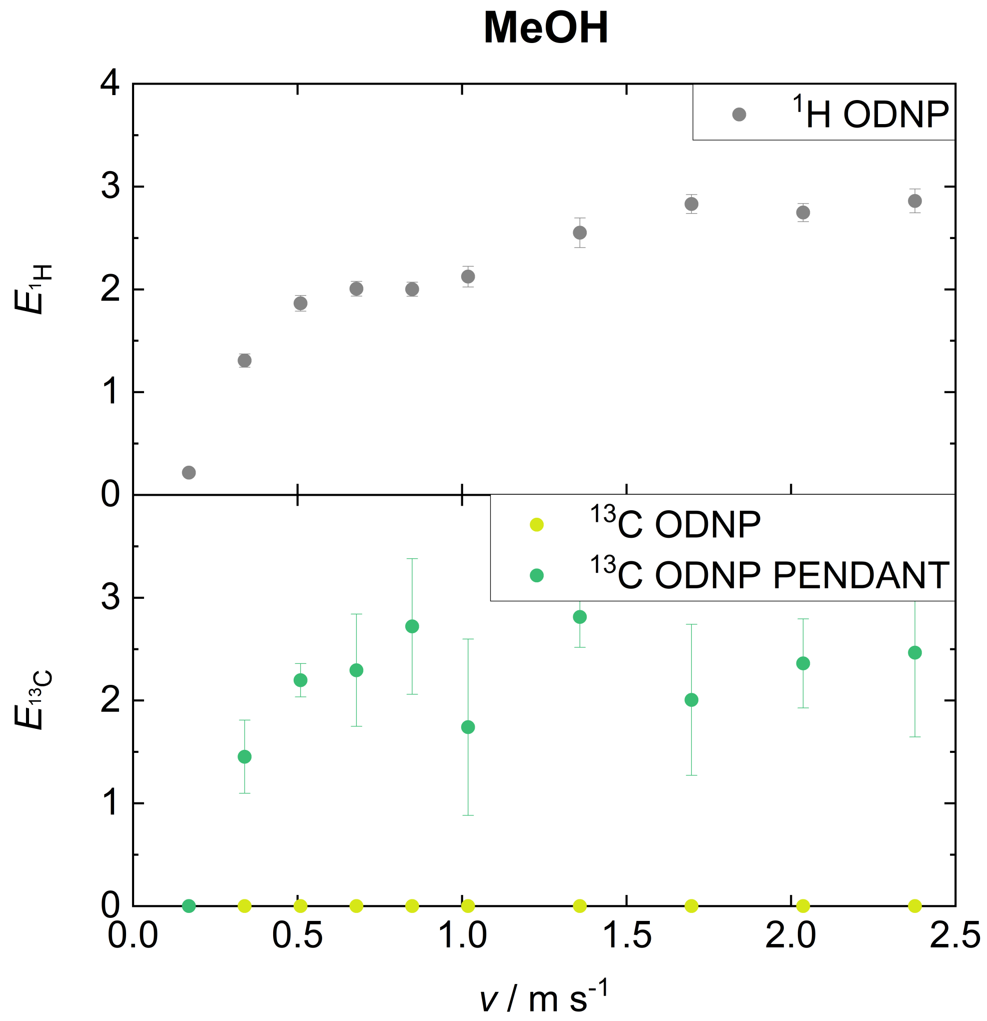

Figure SI.9: Signal enhancements of MeOH (C1) obtained by ^{1}H ODNP, ^{13}C ODNP, and ^{13}C ODNP PENDANT at different flow velocities.
